# Adenine base editing to mimic or correct disease mutations in rodents

**DOI:** 10.1007/s13238-018-0570-3

**Published:** 2018-07-31

**Authors:** Ruotong Ren, Juan Carlos Izpisua Belmonte, Guang-Hui Liu

**Affiliations:** 1 Advanced Innovation Center for Human Brain Protection, National Clinical Research Center for Geriatric Disorders, Xuanwu Hospital of Capital Medical University, Beijing, 100053 China; 2 National Laboratory of Biomacromolecules, CAS Center for Excellence in Biomacromolecules, Institute of Biophysics, Chinese Academy of Sciences, Beijing, 100101 China; 3 University of Chinese Academy of Sciences, Beijing, 100049 China; 4 Gene Expression Laboratory, Salk Institute for Biological Studies, 10010 North Torrey Pines Road, La Jolla, California 92037 USA; 5 Institute of Stem cell and Regeneration, Chinese Academy of Sciences, Beijing, 100101 China

Cytidine base editors (CBEs, rAPOBEC1-nCas9-UGI) and adenine base editors (ABEs, TadA-TadA*-nCas9) are newly developed genome-editing tools that have enabled highly efficient base conversions (C•G to T•A or A•T to G•C) at designated target sites (Komor et al., 2016; Gaudelli et al., 2017). Both types of base editors enzymatically catalyze deamination of target bases and respectively replace C with U or A with I.

After CBEs were first reported, Kim et al. and Liang et al. quickly followed with studies that demonstrated independently the potential of using CBEs to generate point mutation mouse models (Liang et al., 2017a, b; Kim et al., 2017). Liang et al. also showed the effectiveness of CBEs in correcting β-thalassemia point mutations in human embryos (Liang et al., 2017a, b). The advent of ABE has stimulated similarly spirited research efforts. Four groups have now independently shown that ABEs can generate A-to-G point mutation disease mouse and rat models (Ryu et al., 2018; Liu et al., 2018). In addition, Ryu et al. used a viral vector to deliver the ABE into muscles of adult mice so as to correct mutations in the Dunchenne muscular dystrophy (DMD) gene (Ryu et al., 2018). DMD is a progressive and degenerative neuromuscular disease with no effective treatment. The mutation corrected in the study by Ryu et al. was generated by a CBE and not found in DMD patients (Kim et al. 2017). In this issue of *Protein and Cell*, Liang et al. used ABEs to directly link Dunchenne muscular dystrophy with *DMD* gene splice site mutations that have been found in DMD patients (Liang et al., 2018). The three splice site mutations they identified from the DMD mutation database have no cellular or animal models. The group therefore developed the AI-MAST (ABE-induced mRNA splicing defect) method to target specific splice sites of *DMD*. Following delivery into mouse zygotes, the ABE was able to edit the targeted adenine base with high efficiency as was observed in both mouse blastocysts and F0 mice. Moreover, DMD splice site mutant mice displayed obvious muscle weakness and marked upregulation of creatine kinase (CK), which are characteristic symptoms of DMD and indicate a successful one-step generation of DMD disease models using AI-MAST. In addition, the list of potential disease splice site mutations that are targetable by AI-MAST should also prove valuable to researchers studying disease pathogenesis and searching for new therapeutics. Additionally, Yang et al. reported the efficient generation of a hereditary tyrosinemia type I disease model in mice and a glycogen storage disease type II (GSD II; Pompe disease) model in rats using ABE technology in the same issue of *Protein and Cell* (Yang et al., 2018). It is the first report to use base editors to generate disease model in rats. Moreover, they showed that using chemically modified gRNA could improve the editing efficiency of ABE by increasing gRNA stability. Through fusion of adenosine deaminase to SaCas9 and SpCas9 variants, Yang et al. further expanded the targeting scope of ABEs in both cell lines and animals.

Both CBEs and ABEs can in theory precisely generate and/or repair point mutations without inducing DNA double strand breaks (DSBs). However, removal of base U by the uracil DNA glycosylase (UDG) in eukaryotic cells would result in uncontrolled C-to-A/G conversions and DNA DSBs (Liang et al., 2017a, b; Kim et al., 2017; Liu et al., 2018). In contrast, the lack of enzymatic removal of base I would mean potentially more efficient and specific A-to-G conversions by ABEs without detectable DNA DSBs (Ryu et al., 2018; Liu et al., 2018). While the power of ABEs in generating point mutation animal models and correcting disease mutations is clearly evident from the four studies, their specificity remains to be determined, especially in studies using unbiased genome-wide off-target detection platforms. Detection of base editor off-targets differs from that for CRISPR/Cas9 and presents its own challenges. A new methodology that can accurately and efficiently detect genome-wide off-targets of ABEs is therefore sorely needed. Only with such technologies can ABE specificity be properly and effectively improved.
